# Protective effect of fermented *Cyclopia intermedia* against UVB-induced damage in HaCaT human keratinocytes

**DOI:** 10.1186/s12906-016-1218-6

**Published:** 2016-07-29

**Authors:** A-Rang Im, Sung Hum Yeon, Jung Seung Lee, Key An Um, Young -Joon Ahn, Sungwook Chae

**Affiliations:** 1 KM Convergence Research Division, Korea Institute of Oriental Medicine, Daejeon, 305-811 Republic of Korea; 2 Biomodulation Major. Department of Agricultural Biotechnology, Seoul National University, Seoul, 157-742 Republic of Korea; 3 Research Center, Huons Co. Ltd, 55 Hanyangdaehak-ro, Sangrok-gu, Ansan, Gyeonggi-do 426-791 Republic of Korea

**Keywords:** Fermented honeybush extracts, Scale-up fermented honeybush extracts, HaCaT keratinocytes, Matrix metalloproteinase (MMP), Mitogen-activated protein kinase (MAPK), Reactive oxygen species (ROS)

## Abstract

**Background:**

The fermented leaves and stems of *Cyclopia intermedia* are used to brew honeybush tea, a herbal tea indigenous to South Africa. The aim of this study was to evaluate the protective effect of fermented honeybush extracts (FH ex) and scale-up fermented honeybush extracts (SFH ex) against ultraviolet B (UVB)-induced damage in HaCaT keratinocytes. To this end, we examined UVB-induced cell viability, antioxidant enzymes, and inflammatory mediators in HaCaT cells.

**Methods:**

UVB significantly decreased HaCaT cell viability, whereas FH ex and SFH ex did not exhibit cytotoxic effects and increased the viability of the HaCaT cells. To further investigate the protective effects of FH ex on UVB-induced oxidative stress in HaCaT cells, the activities of superoxide dismutase (SOD), catalase (CAT), matrix metalloproteinases (MMPs), pro-inflammatory cytokines and skin barrier function in terms of involucrin, filaggrin, and loricrin were analyzed.

**Results:**

UVB-induced treatment reduced the activity of antioxidant enzymes and skin barrier function, while FH ex or SFH ex increased their activity. These results suggest that FH ex exerted cytoprotective activity against UVB-induced oxidative stress in HaCaT cells through stimulation of antioxidant enzymes activities. Furthermore, FH ex and SFH ex suppressed the UVB-induced expression of inflammatory mediators, such as IL-1β, IL-6, and IL-8, at mRNA level together with down regulation of matrix metalloproteinase (MMPs). In addition, FH ex and SFH ex reversed the phosphorylation of mitogen-activated protein kinase (MAPK) induced by UVB-irradiation. Notably, FH ex and SFH ex markedly inhibited UVB-induced activation of ERK, p38, and JNK. Thus, this agent exhibits anti-oxidative and -inflammatory effects via lowering ROS production, suppressing p38, ERK, and JNK activation, and down-regulating expression of MMPs.

**Conclusions:**

These findings suggest that FH ex and SFH ex can be used as a skin anti-photoaging agent.

## Background

Skin aging has been shown to an intrinsic component and a variable environmental component, termed photoaging. Clinically, photodamaged skin is characterized by loss of elasticity, increased roughness and dryness, irregular pigmentation, and deep wrinkling [1]. Many of the skin changes commonly associated with aging, such as changes in pigmentation, sallowness, and deep wrinkling, are actually the result of sun exposure [2]. In sun-exposed areas, biological processes are superimposed on specific changes that occur in response to UV radiation, including massive elastosis and collagen degeneration [3]. It is important to investigate the effects of biological aging caused by environmental factors, such as exposure to the sun.

Human skin is directly exposed to the air, solar radiation, environmental pollutants, and other mechanical and chemical insults, which are capable of inducing the generation of free radicals, in addition to the reactive oxygen species (ROS) produced by during metabolism [4, 5]. In extrinsic skin aging, the skin suffers additional free radical damage caused by exogenous factors, such as UV exposure; UVA/UVB-radiation contributes to skin aging and photocarcinogenesis [1]. These biological effects of UV-radiation are based on light absorption in chromophores and the subsequent conversion of this energy in chemical reactions. UVB interacts with cellular chromophores and photosensitizers, and the ROS so generated leads to DNA damage and activation of cytoplasmic signal transduction pathways that are related to growth, differentiation, replicative senescence, and connective tissue degradation [2]. Increased ROS generation can overwhelm the antioxidant defense mechanism, resulting in oxidative stress and photodamage of macromolecules and plasma membrane components in the skin [6].

UVB-induced ROS activates matrix metalloproteinases (MMPs) in both the epidermis and dermis, and degrades collagen and elastin in the dermal extracellular matrix [7]. Skin fibroblasts produce MMP-1 in response to UVB irradiation, and keratinocytes play a major role in this process through an indirect paracrine mechanism involving the release of epidermal cytokines after UVB irradiation [8]. UV-induced MMP-1 initiates collagen breakdown by cleaving fibrillar collagen type I and III [9].

UVB exposure of the skin stimulates inflammatory responses via upregulation of pro-inflammatory cytokines, such as tumor necrosis factor-α (TNF-α), interleukin-1β (IL-1β), IL-6, and IL-8 [10]. UVB-induced cell damage leads to activation of ROS-sensitive signaling pathways, such as the mitogen-activated protein kinase (MAPK) pathway [11]. The MAPKs are a family composed of extracellular signal-regulated kinase (ERK), c-Jun NH_2_-terminal kinase (JNK), and p38 MAPK.

Fragments of collagen, a main constituent of the extracellular matrix (ECM), released as a result of MMP-induced collagen cleavage, are bioactive and participate in the regulation of the fibroblast phenotype during photoaging [12]. Collagen has been used as a functional ingredient in skin products due to its efficacy in moisturizing and enhancing elasticity. Moreover, involucrin, filaggrin, and loricrin are major proteins that play an important role in formation of the epidermal skin barrier and skin hydration [13].

H_2_O_2_ is converted to H_2_O and O_2_ either by catalase (CAT), located in the peroxisome as well as in the cytoplasm [14]. In addition to superoxide dismutase (SOD) is another important endogenous antioxidant enzyme that fights against ROS-induced oxidative stress in living organs [15].

The fermented leaves and stems of *Cyclopia intermedia,* or honeybush, is used as a herbal tea indigenous to South Africa [16]. Honeybush is an evergreen shrub belonging to Melianthaceae, which is only distributed in a narrow mountain ridge region along the east–west coast of the Cape region of South Africa, and is highly similar to Rooibos [17]. In previous studies, aqueous extracts of fermented and unfermented honeybush tea have been shown to possess antimutagenic activity against 2-acetylaminofluorene and aflatoxin B1-induced mutagenesis as well as chemoprotective properties against cancer [18]. In our previous study, fermented honeybush extracts (FH ex) showed stronger activity on the inhibition of wrinkle as compared to non-fermented honeybush extracts [19]. FH ex was studied as lab-scale in terms of nonclinical trial using in vitro model. Lab-scale study is giving information for subsequent research for larger scale. FH ex was evaluated as important step in finding feasibility for clinical trial. After developing FH ex in terms of safety and manufacturing cost which is meaningful results for potent clinical intervention, scale-up fermented honeybush extracts (SFH ex) was established.

In this study, we investigated the signaling pathway accounting for the cytoprotective effects of FH ex and SFH ex against UVB-induced oxidative stress in HaCaT cells. We found that FH ex or SFH ex protected cells against UVB-induced oxidative stress through stimulation of antioxidant enzyme activities. Furthermore, FH ex and SFH ex suppressed UVB-induced expression of inflammatory mediators, such as IL-1β, IL-6, and IL-8, at mRNA level, suppressing p38, ERK and JNK activation together with MMPs expression.

## Methods

### Materials

*Cyclopia intermedia* was purchased from Rooibos.Ltd (www.rooibosltd.co.za), and dried samples (1.0 kg) were extracted with distilled water (8 L) two times under reflux for 1 h. The filtrate was evaporated in vacuo to afford the extract (200 g). To prepare fermented honeybush (FH ex), the method was slightly modied from the previous study [19]. To prepare FH ex, 1.0 % of lactic acid bacteria (*Streptococcus thermophilus*) was inoculated in 100 mL of water with 5 % of the honeybush extract, 12.6 % of whole milk powder, and 3 % of sucrose. The mixture was fermented in a 37 °C incubator for 24 h. For the preparation of scale-up fermented honyebush (SFH ex), honeybush was extracted with water 2 times under reflux and then filtered. The filtrate was evaporated to give 20 brix extract. To prepare SFH ex, 10 % of lactic acid bacteria (*Streptococcus thermophilus*) was inoculated in 0.5 % of whole milk powder with 3 % of lactose, 23 % of the honeybush extract (20 brix), and 63.5 % of purified water. The mixture was fermented in a 37 °C incubator for 48 h. After sterilization of fermented mixture, final product was obtained by mixing dextrin (DE12) and spray drying. DMEM media and fetal bovine serum (FBS) were purchased from Gibco BRL (Grand Island, NY). A CellTiter Aqueous One Solution Cell Proliferation Assay kit (MTS) was purchased from Promega Co. (Madison, WI). Human total MMP-1 and -9 ELISA kits were obtained from R&D Systems, Inc. (Minneapolis, MN).

### Cell culture and UVB irradiation

HaCaT cells, an immortalized non-tumorigenic human keratinocyte cell line obtained from Huons Co. Ltd, (Gyeonggi-do, Korea), were maintained in Dulbecco’s modified Eagle’s medium (DMEM) supplemented with 10 % fetal bovine serum and 1 % antibiotics at 37 °C and 5 % CO_2_ in a humidified incubator. HaCaT cells were seeded and allowed to adhere for 24 h. The cells were treated with various concentrations of FH ex and SFH ex and exposed to UVB radiation at a dose of 20 mJ/cm^2^. Cells that received no pretreatment and were not exposed to UVB irradiation served as the control.

### Cell viability assay

HaCaT cells (1 × 10^4^) were seeded in 96-well culture plates and treated with various concentrations of FH ex and SFH ex for 24 h. Before UVB treatment, the cell culture medium was replaced by phosphate-buffered saline (PBS) and exposed to 20 mJ/cm^2^ of UVB. Immediately after UVB irradiation, cell viability was assessed after 1 h by reduction of [3-(4,5-dimethylthiazol-2-yl)-5-(3-carboxymethoxyphenyl)-2-(4-sulfophenyl)-2H-tetrazolium, inner salt (MTS) to formazan, according to the manufacturer’s instruction. Samples were then assayed at 490 nm using a microplate fluorimeter (Molecular Devices, Sunnyvale, CA).

### Antioxidant enzymes activities

SOD and CAT activity was measured using a colorimetric assay kit (Cayman Chemical Co., Ann Arbor, MI) according to the manufacturer’s protocol. Cells were treated with FH ex for 24 h. After UVB exposure, cells were homogenized in cold lysis buffer. The absorbance was measured at 450 nm to determine SOD activity, and at 540 nm to determine CAT activity, by using a plate reader (Molecular Devices, Sunnyvale, CA).

### Determination of MMP-1 and-9 secretions by ELISA

After UVB irradiation, matrix metalloproteinases-1 and -9 levels in the culture media of HaCaT cells (5 × 10^4^) were determined using human total MMP-1 and -9 ELISA kits according to the manufacturer’s instructions. HaCaT cells were seeded in 96-well plates and treated with FH ex and SFH ex. After exposure to UVB, the culture supernatant was collected and centrifuged at 13,000 rpm for 5 min. The levels of MMP-1 and MMP-9 in culture supernatants were quantified by colorimetric analysis.

### Quantitative Real-time PCR assays

Total RNA was extracted from each sample treated with UVB using TRIzol reagent (Invitrogen, Carlsbad, CA). Real-time PCR was performed using TaqMan Gene assays (Applied Biosystems, Foster City, CA) specific for the genes encoding MMP-1, MMP-9, tissue inhibitor of metalloproteinase (TIMP), involucrin, filaggrin, loricrin, IL-1β, IL-6, IL-8, on a QuantStudio^TM^ 6 Flex Real-Time PCR systems. Relative amounts of cDNA were calculated by the relative quantification (ΔΔCt) method. Each sample was run in triplicate and the gene encoding β-actin was used as a control to normalize for differences in the amount of total RNA in each sample.

### Western blot

Protein was extracted from HaCaT cells. Twenty microgram of protein lysates from each sample were electrophoresed on a 10 % sodium dodecyl sulfate (SDS)-polyacrylamide gel and were then transferred to PVDF membranes. Membranes were blocked for 1 h at room temperature in 5 % skim milk solution. Blots were incubated overnight at 4 °C with a 1:1000-diluted monoclonal antibody. The blots were washed three times for 10 min each time with Tris-buffered Saline (TBST). The membranes were then incubated for 2 h with secondary antibody. Proteins were detected using an enhanced chemiluminescence.

### Statistics

Results were compared using Tukey’s multiple comparison test, where a *p* < 0.05 was considered statistically significant. GraphPad Prism 5 was used for statistical analyses.

## Results

### Protective effects of FH ex and SFH ex against UVB-induced HaCaT cell damage

**Fig. 1:**
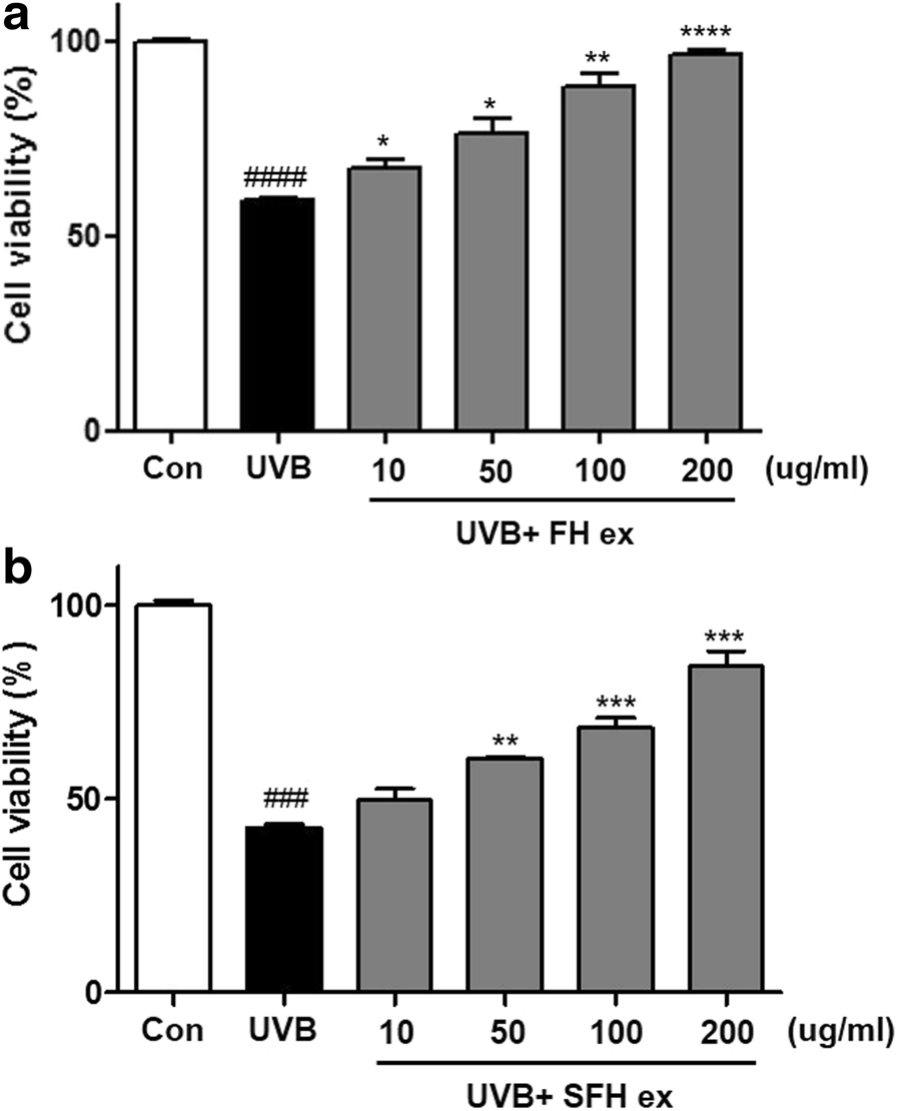
Cell viability of human keratinocytes after UVB exposure. HaCaT cells were pretreated with various concentrations of (**a**) fermented honeybush extract (FH ex) and (**b**) scale up fermented honeybush extract (SFH ex) for 24 h, followed by UVB irradiation. Data are reported as percentages of the control group. ####* P* < 0.0001 and ### *P*< 0.001 as percentages of the control group. ****P < 0.0001, ***P < 0.001, **P < 0.01 and *P < 0.05 *vs* UVB vehicle group

We investigated the effect of FH ex and SFH ex on the proliferation of HaCaT cells after exposure to UVB. Cell viability was reduced to 59.14 % by UVB irradiation in the absence of FH ex, but cell proliferation was increased up to 96.78 % (Fig. 1a) in its presence. Also, in SFH ex group, cell proliferation was increased up to 84.07 % (Fig. 1b) in its presence.

### Effects of FH ex on the activity of antioxidant enzymes in UVB-induced HaCaT cells

**Fig. 2:**
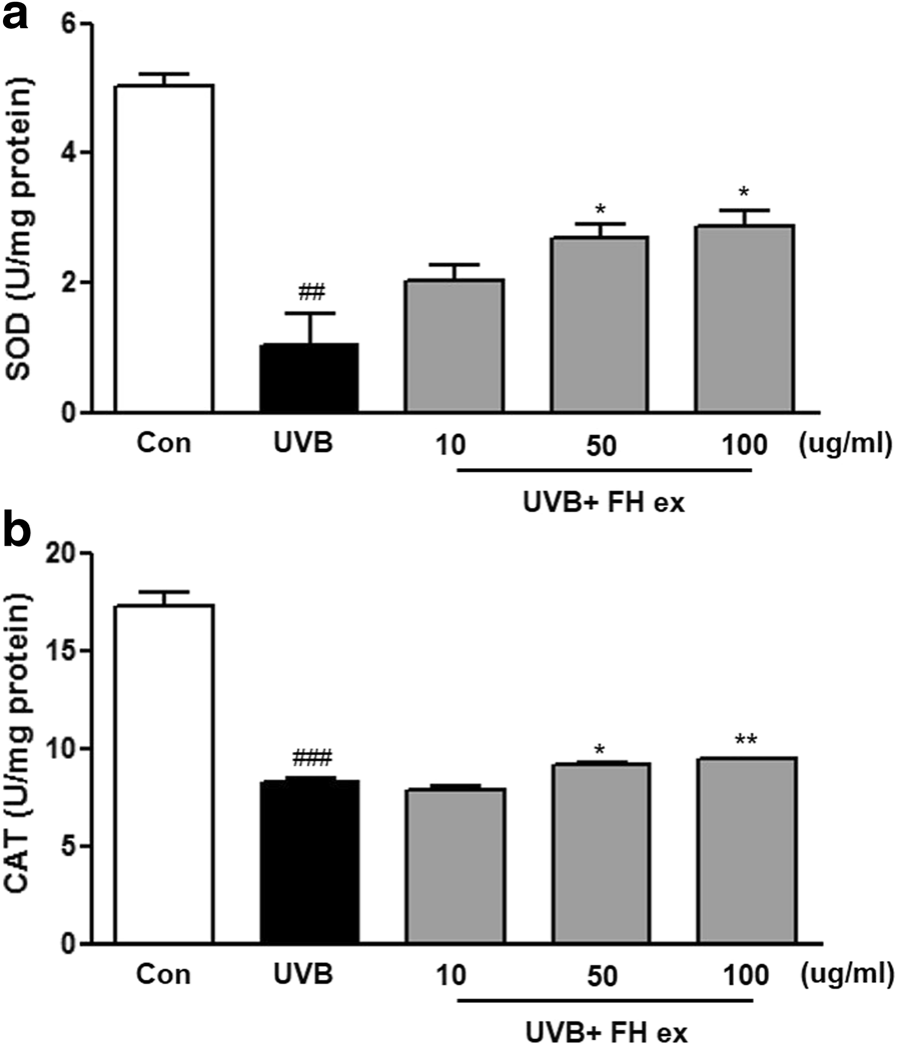
Effects of FH ex on the activity of (**a**) SOD, and (**b**) CAT in HaCaT cells exposed to UVB. ### *P* < 0.001, and ## *P* < 0.01 *vs* control group. **P < 0.01 and *P < 0.05 *vs* UVB vehicle group

To investigate whether the radical scavenging activity of FH ex was mediated by antioxidant enzymes, the activities of antioxidant enzymes were examined in HaCaT cells after UVB exposure. SOD activity decreased to 0.7 U/ml in UVB-induced cells compared to the untreated control (Fig. 2a), SOD activity was enhanced by FH ex treatment in a dose-dependent manner. Furthermore, in comparison with the control group, the activities of CAT in the UVB-induced group were decreased, but treatment with FH ex increased CAT activities (Fig. 2b). These results revealed that FH ex could protect the activities of antioxidant enzymes that scavenge free radicals and thereby could further inhibit UVB-induced oxidative stress.

### Effects of FH ex and SFH ex on UVB-induced secretion of MMP-1 and MMP-9

**Fig. 3:**
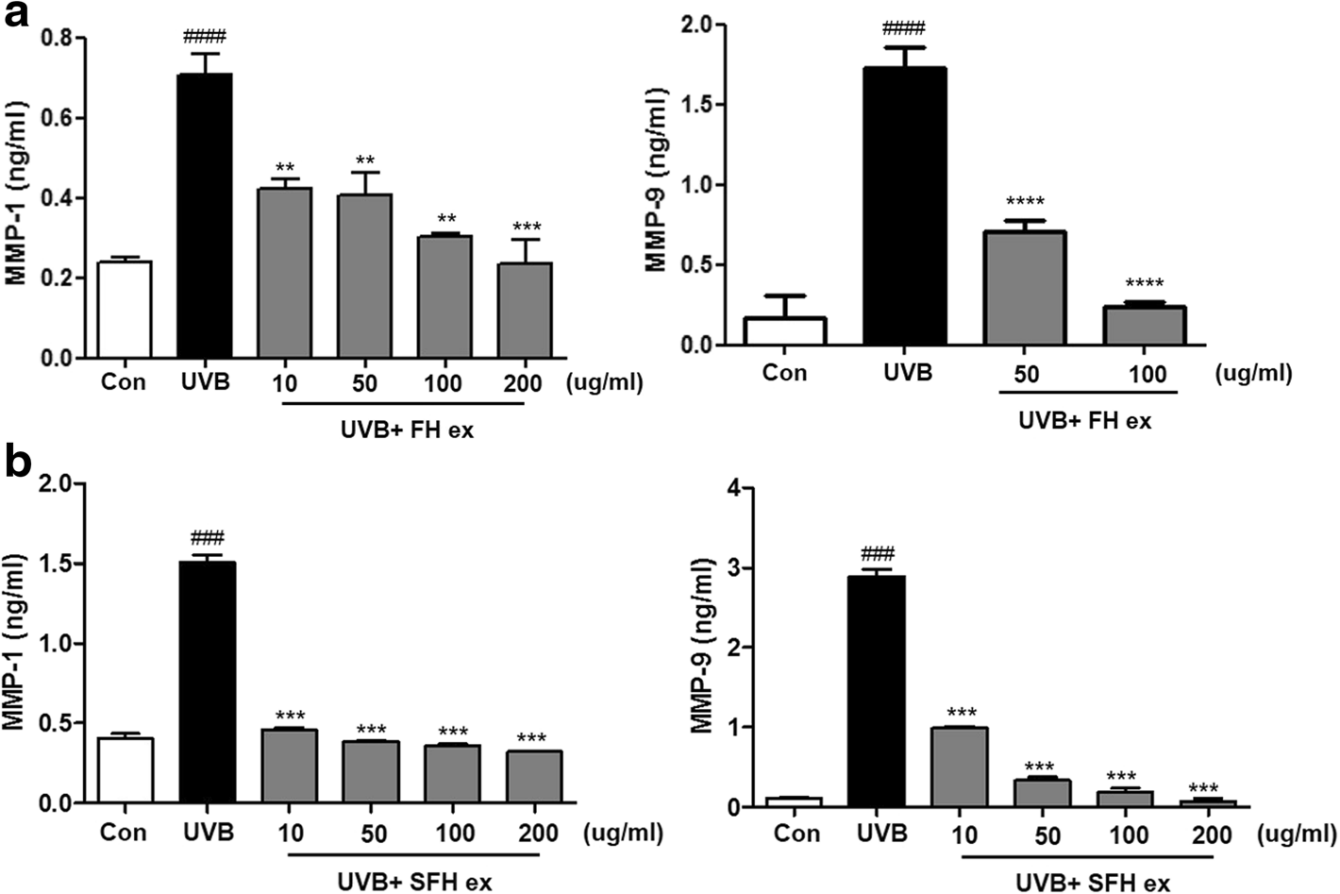
Effects of (**a**) FH ex and (**b**) SFH ex on MMP-1, and MMP-9 in UVB-induced HaCaT cells. Cells were treated with FH ex and SFH ex for 24 h before UVB irradiation. ELISA analyses of MMP-1 and −9 levels in cultured medium after UVB irradiation. #### *P* < 0.0001 and ### *P* < 0.001 *vs* control group. ****P < 0.0001, ***P < 0.001, and **P < 0.01 *vs* UVB vehicle group

We evaluated the effect of FH ex and SFH ex on UVB-induced MMP expression in HaCaT cells. UVB irradiation of HaCaT cells resulted in a marked increase in MMP-1 and -9 levels (Fig. 3). ELISA analysis revealed that FH ex and SFH ex treatment reduced MMP-1 and -9 protein levels in the culture media of HaCaT cells in a dose-dependent manner.

### Effects of FH ex and SFH ex on UVB-induced secretion of MMP, TIMP, and procollagen

**Fig. 4:**
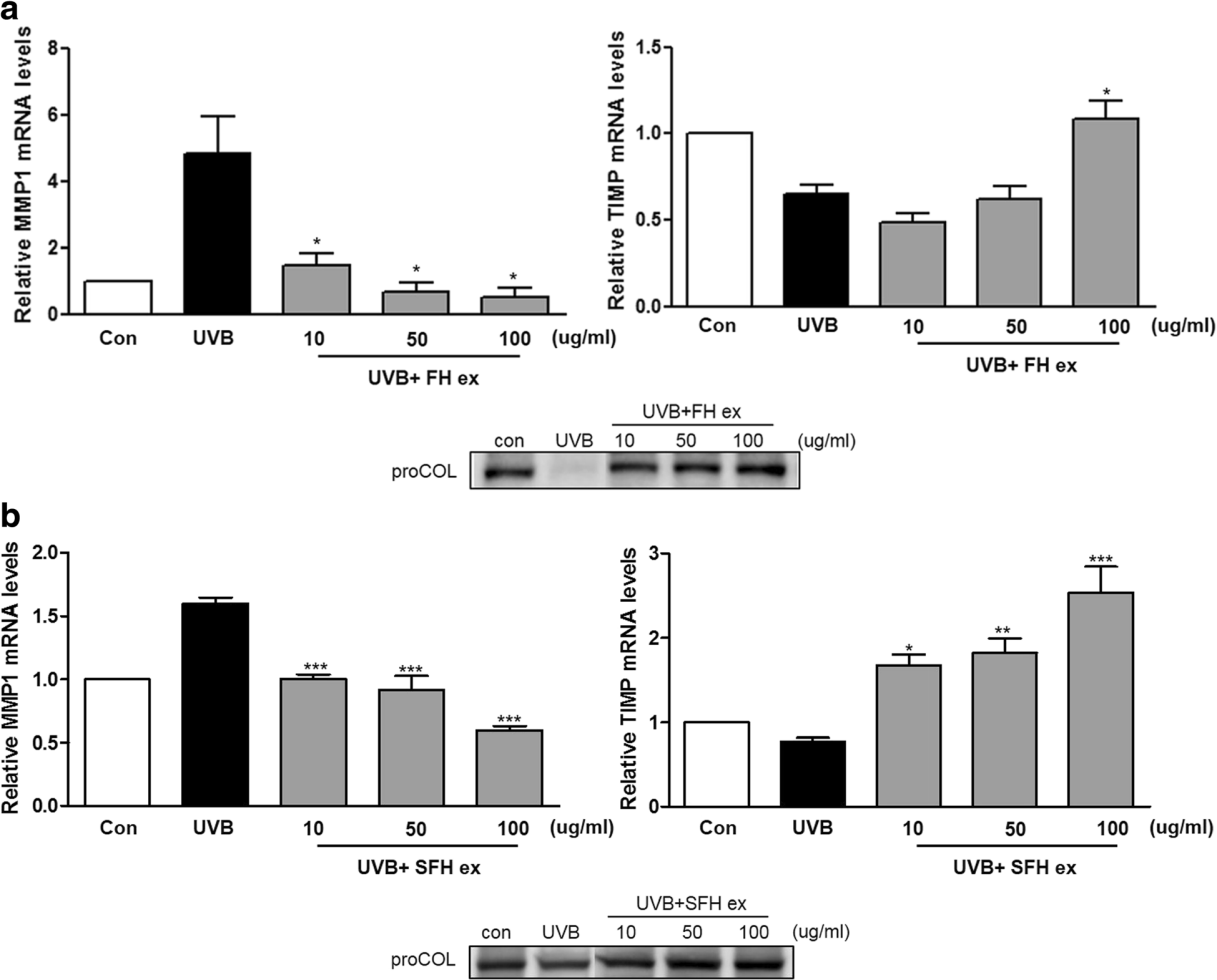
(**a**) FH ex and (**b**) SFH ex treatment attenuates UVB-induced MMP and increases TIMP levels in HaCaT cells. Total RNA was extracted from HaCaT cells. mRNA levels of MMP-1, and TIMPs were determined by RT-PCR analysis. Pro-collagen protein levels were determined by western blot analysis. *** *P*< 0.001, **P < 0.01, *P < 0.05 *vs* UVB vehicle group

The mRNA expression of MMP-1 and TIMP genes in HaCaT cells after UVB irradiation was measured by RT-PCR (Fig. 4). UVB caused a decrease in TIMP-1 mRNA levels, whereas pretreatment with FH ex and SFH ex inhibited this decrease. Western blotting demonstrated that UVB irradiation reduced the level of procollagen expression compared with the control group (Fig. 4), and pretreatment with FH ex and SFH ex prevented this decrease.

### Effects of FH ex and SFH ex on involucrin, filaggrin, and loricrin levels in UVB- induced HaCaT cells

**Fig. 5:**
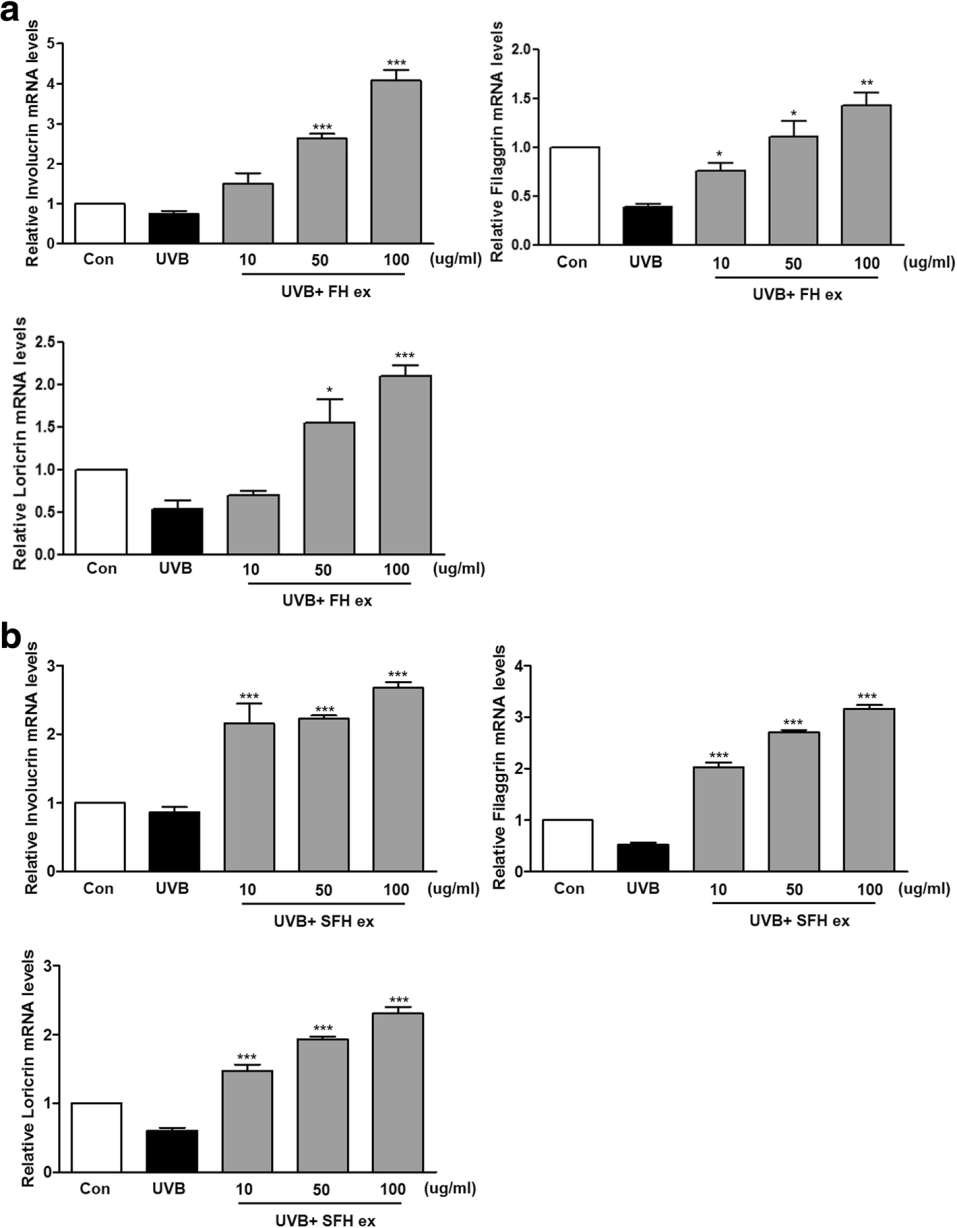
Effects (**a**) FH ex and (**b**) SFH ex on involucrin, filaggrin, and loricrin levels in UVB-induced HaCaT cells. ***P < 0.001, **P < 0.01, and *P < 0.05 *vs* UVB vehicle group

We showed that the expression of involucrin, filaggrin, and loricrin was decreased by UVB. As shown in Fig. 5, mRNA expression of involucrin, filaggrin, and loricrin in UVB-induced cells treated with FH ex and SFH ex was increased, as compared to UVB exposed cells.

### Effects of FH ex and SFH ex on IL-1β, IL-6, and IL-8 levels in UVB-induced HaCaT cells

**Fig. 6:**
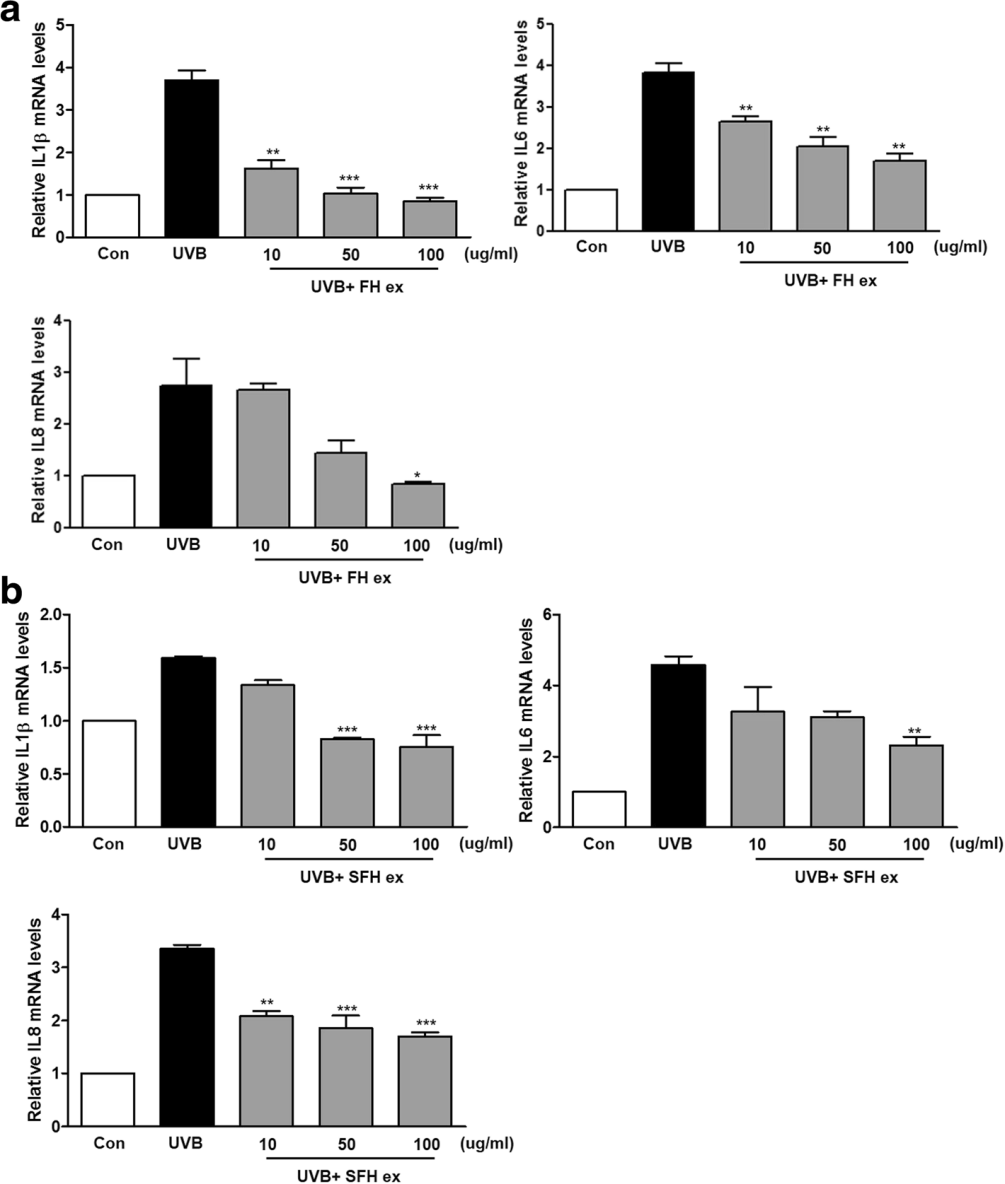
Effects (**a**) FH ex and (**b**) SFH ex on the pro-inflammatory cytokines of UVB-induced HaCaT cells. IL-1β, IL-6, and IL-8 secretion mRNA were determined by RT-PCR analysis. ***P < 0.001, **P < 0.01, and *P < 0.05 *vs* UVB vehicle group

To evaluate the effects of FH ex and SFH ex on the UVB-induced production of inflammatory mediators, cells were treated with FH ex and SFH ex for 24 h prior to UVB exposure. UVB significantly increased the levels of IL-1β, IL-6, and IL-8 mRNA as compared with those in the normal cells (Fig. 6), but FH ex and SFH ex inhibited this elevation. These results suggested that the protective effect of FH ex and SFH ex may be partly associated with inhibition of the release of pro-inflammatory cytokines in response to UVB induction.

### Effects of FH ex and SFH ex on phosphorylation of MAPKs in UVB-induced HaCaT cells

**Fig. 7:**
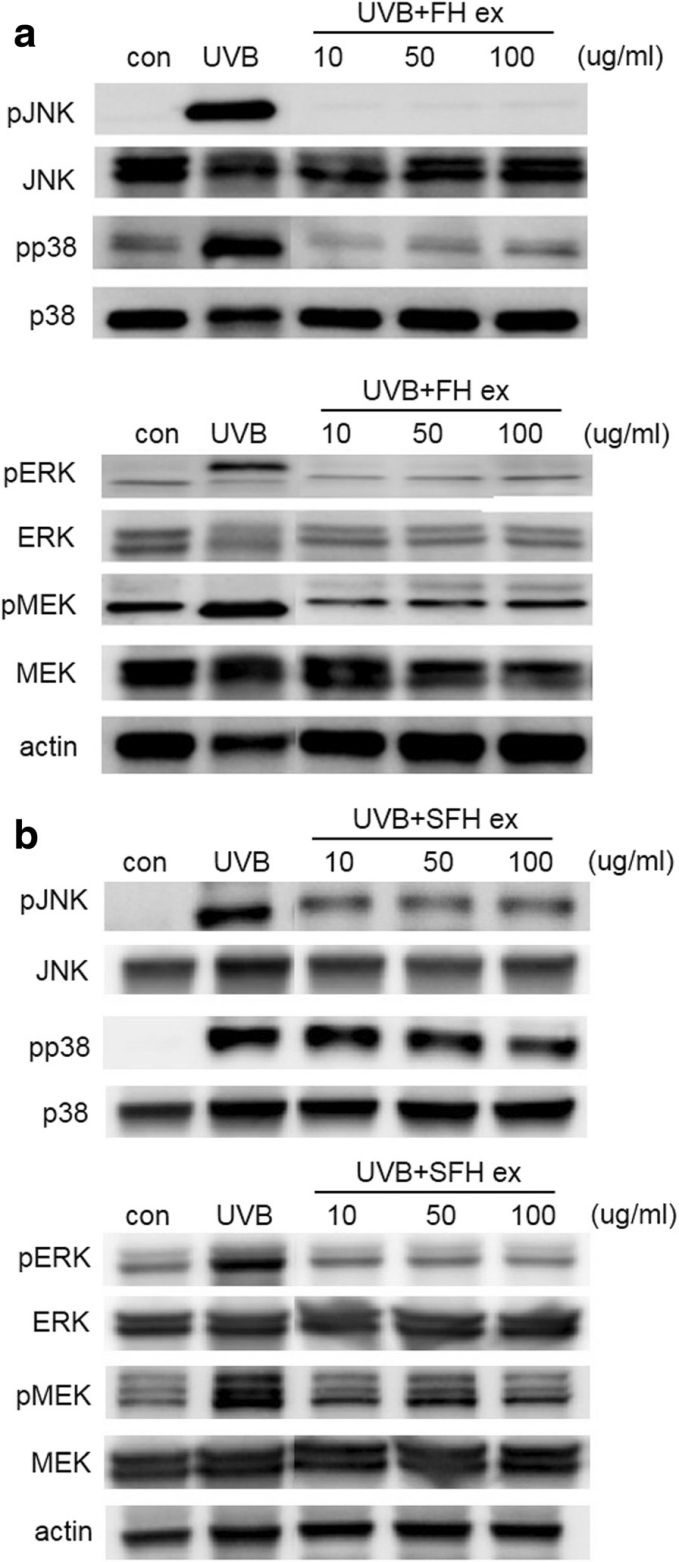
Inhibitory effects of (**a**) FH ex and (**b**) SFH ex on UVB-induced phosphorylation of ERK, MEK, JNK, and p38 in HaCaT cells. Phospho-ERK, phosphor-MEK, phospho-p38, phospho-JNK, and β-actin protein levels were determined by western blot analysis

We examined whether FH ex and SFH ex could suppress UVB-induced phosphorylation of MAPKs, including ERK, MEK, p38, and JNK. As shown in Fig. 7, irradiation of UVB resulted in phosphorylation of ERK, MEK, p38, and JNK, whereas treatment of cells with FH ex and SFH ex prior to UVB irradiation inhibited the UVB-mediated phosphorylation of these MAPKs.

## Discussion

Herbal ingredients are used for dermatological purposes, as oral dietary supplements or are incorporated into topical formulations [20]. Their potential antioxidant and anti-inflammatory benefits may prove beneficial for a number of conditions that dermatologists routinely treat, such as rosacea, photoaging, and skin cancer [21]. For example, topical application of green and white tea extracts can reduce some of the detrimental immunomodulatory effects of photodamage [22]. FH ex was prepared for the evaluation of feasibility to examine the scale-up study. Pilot studies are mainly focused on the efficacy rather than safety and production cost, however it can inform modifications for the design of larger scale study. A pilot study provides opportunity to enhance data integrity and the protection of human subjects in terms of toxicology [23]. FH ex used in our paper was studied as pilot scale prior to clinical research manufactured in a larger facility. As our data showed FH ex exerted cytoprotective activity against UVB-induced oxidative stress in HaCaT cells, FH ex was modified for human clinical trial and commercial requirements. After evaluation of SFH ex regarding toxicology studies using SD rat and beagle in GLP facility (data not shown), the SFH ex was established and tested for skin damage. In our results, biological activity regarding cytoprotective effects of SFH ex showed similar level as FH ex to fit novel intervention for clinical trials. Taken together SFH ex can be applied for clinical trials to reduce the risk of toxicity and manufacturing cost.

Human skin is equipped with a network of antioxidant enzymes that play an important role in the balance of intracellular ROS levels [24]. Exposure of human or mouse skin to UVB radiation results in excessive generation of ROS that overwhelms the antioxidant defense system, resulting in oxidative stress [4]. ROS in the biological system have been linked to skin cancers, DNA damage, cutaneous aging, and many inflammatory disorders. In a previous study, it has been shown that UV light induced decreases in the antioxidant enzymes CAT and SOD [25]. In this study, FH ex could protect the activities of antioxidant enzymes that scavenge free radicals such as SOD and CAT and thereby could further inhibit UVB-induced oxidative stress.

MMPs are a large family of degradative enzymes that play important roles in tissue destruction during pathological processes, such as arthritis, skin aging, tumor invasion, and metastasis [26]. MMPs degrade collagen fibers in the skin, which form an important part of the connective tissue that is involved in the maintenance of dermal strength and elasticity [27]. And TIMPs are primary enzymes involved in the regulation of collagen metabolism; TIMP-1 inhibits the activity of MMPs that relative to TIMPs suggests that MMPs activities are elevated in photodamaged skin resulted in breakdown of collagen [28]. Irradiation of human skin with even a single dose of UV light has been shown to increase the activities of MMPs, which has been associated with significant degradation of collagen fibers [29]. A decrease in procollagen expression caused by repeated UV irradiation is considered to be a cause of photoaging [30]. The present study found that treatment of HaCaT cells with FH ex and SFH ex prior to UVB irradiation significantly decreased MMP-1 and MMP-9 expression, and increased that of TIMP-1.

Filaggrin and profilaggrin are essential for maintaining homeostasis in the epidermis and filaggrin also undergoes further processing in the upper stratum corneum to release free amino acids that assist in water retention [31]. Involucrin, a differentiation marker normally expressed by irreversibly differentiated keratinocytes in the stratum corneum [32]. Filaggrin, involucrin, and loricrin are important source for maintaining skin moisture [33]. In a previous study, downregulation of filaggrin and loricrin have been shown to be involved in skin reconstructed in vitro after UVB exposure [34]. In this study, mRNA expression of involucrin, filaggrin, and loricrin in UVB-induced cells treated with FH ex and SFH ex was increased, as compared to UVB exposed cells. These results showed that FH ex and SFH ex have a skin hydration effects.

Keratinocytes respond to the major changes in inflammation and immunomodulation observed after UVB exposure, at least in part via UVB-induced expression of inflammatory enzymes and release of pro-inflammatory mediators [35]. Chronic UVB irradiation was shown to increase the production of some pro-inflammatory cytokines, such as TNF-α, IL-1β, IL-6, and IL-8, leading to cell death in HaCaT cells [36]. We here examined the effect of FH ex and SFH ex on UVB-induced pro-inflammatory cytokine production in HaCaT cells and found that the inhibitory effect of FH ex and SFH ex on the production of inflammatory mediators was accompanied by reduced mRNA expression levels of IL-1β, IL-6, and IL-8.

ROS are known to play a role in UV-induced skin damage and aging. Exposure to UV irradiation induces the production of ROS in skin cells; this ROS activates cellular signaling pathways that activate kinases such as p38, JNK, and MAPK. Previous studies have shown activation of the three MAP kinases, ERK, JNK, and p38, within 1 h of acute UV radiation [37]. We observed that FH ex and SFH ex inhibited UVB-induced activation of JNK, p38, pMEK and ERK.

## Conclusion

In conclusion, these agents exhibit anti-oxidative and inflammatory effects that are mediated by lowering ROS production, suppressing p38, ERK, MEK and JNK activation, and down-regulating expression of MMPs. These findings suggest that FH ex and SFH ex can be used as a skin anti-photoaging agent.

## Abbreviations

CAT, catalase; DMEM, Dulbecco's modified eagle medium; FH ex, fermented honeybush extracts; MAPK, mitogen-activated protein kinase; MMPs, matrix metalloproteinase; mRNA, messenger RNA; PBS, phosphate-buffered saline; PVDF, polyvinylidene difluoride; qRT-PCR, quantitative real-time polymerase chain reaction; RNA, ribonucleic acid; ROS, reactive oxygen species; SFH ex, scale-up fermented honeybush extracts; SOD, superoxide dismutase; TBST, Tris-buffered Saline; TIMP, tissue inhibitor of metalloproteinase; UVB, ultraviolet B.
